# Development and implementation of a Type I-C CRISPR-based programmable repression system for *Neisseria gonorrhoeae*

**DOI:** 10.1128/mbio.03025-23

**Published:** 2023-12-21

**Authors:** Wendy E. Geslewitz, Amaris Cardenas, Xufei Zhou, Yan Zhang, Alison K. Criss, H Steven Seifert

**Affiliations:** 1 Department of Microbiology and Immunology, Northwestern University, Chicago, Illinois, USA; 2 Department of Microbiology, Immunology, and Cancer Biology, University of Virginia, Charlottesville, Virginia, USA; 3 Department of Biological Chemistry, University of Michigan, Ann Arbor, Michigan, USA; McGovern Medical School, Houston, Texas, USA

**Keywords:** CRISPR, *Neisseria gonorrhoeae*, Opa, neutrophils, essential genes, pili, transcriptional regulation

## Abstract

Clustered regularly interspaced short palindromic repeats (CRISPR) are prokaryotic adaptive immune systems regularly utilized as DNA-editing tools. While *Neisseria gonorrhoeae* does not have an endogenous CRISPR, the commensal species *Neisseria lactamica* encodes a functional Type I-C CRISPR-Cas system. We have established an isopropyl β-d-1-thiogalactopyranoside added (IPTG)-inducible, CRISPR interference (CRISPRi) platform based on the *N. lactamica* Type I-C CRISPR missing the Cas3 nuclease to allow locus-specific transcriptional repression. As proof of principle, we targeted a non-phase-variable version of the *opaD* gene. We show that CRISPRi can downregulate *opaD* gene and protein expression, resulting in bacterial inability to stimulate neutrophil oxidative responses and to bind to an N-terminal fragment of CEACAM1. Importantly, we used CRISPRi to effectively knockdown all the transcripts of all 11 *opa* genes using a five-spacer CRISPR array, allowing control of the entire phase-variable *opa* family in strain FA1090. We also report that repression is reversible following IPTG removal. Finally, we showed that the Type I-C CRISPRi system can conditionally reduce the expression of two essential genes. This CRISPRi system will allow the interrogation of every Gc gene, essential and non-essential, to study physiology and pathogenesis and aid in antimicrobial development.

**IMPORTANCE:**

Clustered regularly interspaced short palindromic repeats (CRISPR)-Cas systems have proven instrumental in genetically manipulating many eukaryotic and prokaryotic organisms. Despite its usefulness, a CRISPR system had yet to be developed for use in *Neisseria gonorrhoeae* (Gc), a bacterium that is the main etiological agent of gonorrhea infection. Here, we developed a programmable and IPTG-inducible Type I-C CRISPR interference (CRISPRi) system derived from the commensal species *Neisseria lactamica* as a gene repression system in Gc. As opposed to generating genetic knockouts, the Type I-C CRISPRi system allows us to block transcription of specific genes without generating deletions in the DNA. We explored the properties of this system and found that a minimal spacer array is sufficient for gene repression while also facilitating efficient spacer reprogramming. Importantly, we also show that we can use CRISPRi to knockdown genes that are essential to Gc that cannot normally be knocked out under laboratory settings. Gc encodes ~800 essential genes, many of which have no predicted function. We predict that this Type I-C CRISPRi system can be used to help categorize gene functions and perhaps contribute to the development of novel therapeutics for gonorrhea.

## INTRODUCTION

Gonorrhea represents a major public health burden and is estimated to affect ~87 million individuals worldwide (1). The main causative agent of gonorrhea is the bacterium *N. gonorrhoeae* (gonococcus, Gc), an obligate human pathogen. Gc colonizes mucosal surfaces such as the urogenital tract, anal, ocular, and pharyngeal mucosa (2–5). In rare circumstances, Gc exits the genital tract and causes disseminated gonococcal infection which may lead to arthritis and endocarditis (6). Generating effective vaccines to prevent Gc is difficult, as the bacteria undergo extensive phase and antigenic variation, allowing for the creation of variant antigens to avoid immune recognition (7). Treatment of Gc infection is further hampered by its development of antibiotic resistance; the bacteria are resistant to most antibiotics, and new strains have displayed resistance to the first-line dual therapy treatments of azithromycin and ceftriaxone (8–11).

The most common way to study Gc gene function is by generating loss-of-function mutations. However, essential genes cannot be deleted and are important as possible targets for antimicrobials or vaccines. Gc is predicted to possess about 800 essential genes, of which 130 have no predicted function (12, 13). It is possible to overcome the inability to delete essential genes by inactivating the gene of interest while regulating its expression at an ectopic locus (14, 15). This approach has issues with achieving wild-type levels of gene expression and the inherent leakiness of transgene expression. Alternatively, temperature-sensitive mutations can create conditionally lethal strains where an essential gene is non-functional at certain temperatures (16). However, Gc is restricted to narrow growth temperatures (17, 18), making temperature-sensitive mutations difficult to isolate. Gene overexpression is another mechanism to study essential genes (19, 20), but there is potential for toxicity associated with overexpression and artifactual phenotypes (21, 22).

The genetic modulation of many prokaryotic organisms relies on engineered clustered regularly interspaced short palindromic repeats (CRISPR)-Cas systems. CRISPR is an adaptive immune system encoded by about half of all bacterial species (23). These systems are designed to recognize and interfere with previously encountered DNA, particularly from bacteriophages (24, 25). CRISPR is comprised of two key components: the CRISPR array and the effector Cas enzymes (26). The CRISPR array is a genetic locus in which short sequences from past invading genetic elements (spacers) are separated by repeated palindromic sequences (repeats) (25). In many CRISPR-Cas systems, the sequence of the spacer in the targeted DNA (protospacer) is adjacent to the protospacer adjacent motif (PAM), a short DNA sequence that allows for the differentiation between the target and the CRISPR array (27). The transcribed and processed spacer (crRNA) complexes with the Cas effector enzyme to guide it and cleave the complementary target DNA. The specificity of CRISPR-directed localization in the bacterial genome has led to it being co-opted for various purposes, including generating mutations (28), activating gene expression (29), and gene silencing (30). Despite the usefulness of these molecular tools, an engineered CRISPR-Cas system has not yet been developed in Gc.

Here, we present the development of a novel, inducible, minimal CRISPR interference (CRISPRi) system for gene-specific transcription repression in Gc, which lacks its own functional CRISPR-Cas system (31). We introduced the DNA-binding module of the Type I-C CRISPR-Cas from *Neisseria lactamica* (Nla)—a commensal *Neisseria* species closely related to Gc (32)—to a transcriptionally silent intergenic region within the Gc chromosome, under the control of an isopropyl β-d-1-thiogalactopyranoside (IPTG)-driven promoter (33, 34). The Type I CRISPR is the most abundant and diverse type of CRISPR in microbes (23) and has been engineered for genome modulation in many bacterial species (35–37). These systems typically use a multi-protein complex called Cascade (the CRISPR-associated complex for antiviral defense) (38) for RNA-guided binding and cleavage of the target DNA and a nuclease-helicase Cas3 for processive DNA degradation. We harnessed the Cascade complex (without the Cas3 nuclease-helicase) of Nla I-C CRISPR into a programmable gene repression system. By reprogramming Nla Cascade to bind a specific DNA sequence to obstruct RNA polymerase access, we show that the CRISPRi system can repress the expression of individual Gc genes and an entire gene family. Ectopic expression of Nla Cascade in Gc results in a small fitness cost independent of having a guide RNA sequence. We also show that the CRISPRi system can enable inducible repression of essential genes to create conditional lethal strains. The Nla Type I-C CRISPRi system presents as a versatile system for genetic manipulation in Gc.

## RESULTS

### Introduction of the *N. lactamica* Type I-C CRISPRi construct into Gc

**Fig 1:**
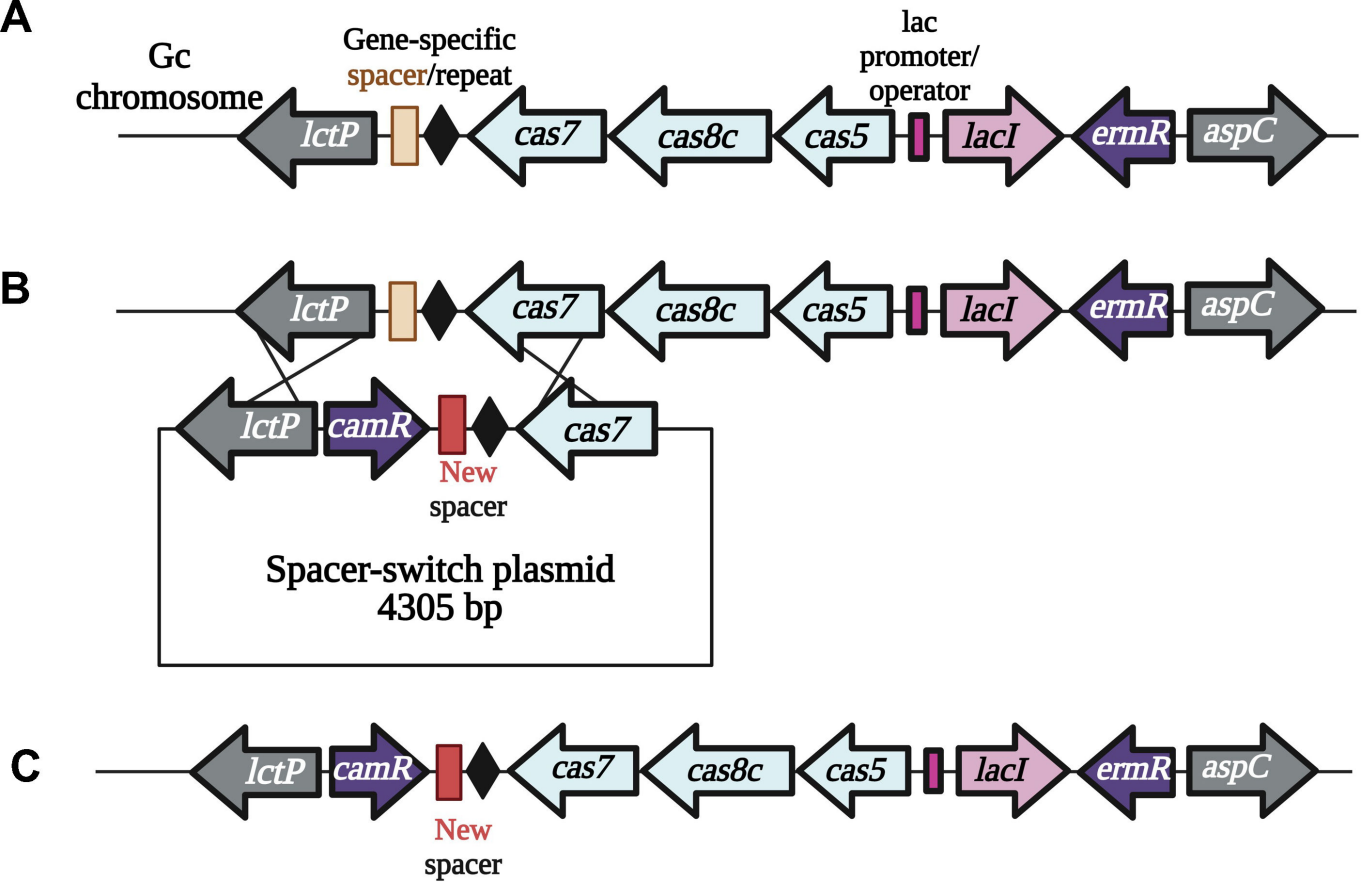
Schematics of the *Neisseria lactamica*-derived Type I-C CRISPRi system. (**A**) Schematic of the CRISPRi genetic locus after recombination of the vector into the Gc chromosome. (**B**) Schematic demonstrating recombination of the spacer switch plasmid containing the replacement spacer (black outlined box) into the CRISPRi locus of the Gc chromosome. (**C**) Diagram of the CRISPRi locus with the replaced spacer.

We designed the original Type I-C CRISPRi delivery vector to introduce components of the *N. lactamica* Type I-C Cascade system into the Gc genome using DNA transformation (Fig. 1A). This vector encodes the Cascade complex (*cas5*, *cas8c*, *cas7*, internal *cas11* embedded within *cas8*, and a CRISPR array) adjacent to the *lac* regulatory sequences (*lacI* and the *lac* promoter/operator) (38), allowing for IPTG regulation of Cascade expression. The Nla I-C CRISPR array contains a 32 base pair (bp) repeat, 35 bp gene-specific spacer, and the leader sequence. The Type I-C CRISPRi delivery vector lacks the *cas3* gene, rendering this an interference system. The delivery vector encodes an erythromycin resistance cassette to select transformants that integrated the Type I-C CRISPRi system into the Gc chromosome using the flanking homology to the *lctP* and *aspC* genes in the chromosomal complementation plasmid pGCC4 (39) (Fig. 1B). However, the transformation efficiency of the Type I-C CRISPRi construct into Gc was low (~3 × 10^−8^ transformants/CFU).

To allow for ease of cloning, we generated a minimal single-repeat, single-spacer (R-S) as opposed to the repeat-spacer-repeat (R-S-R) array. We found that the R-S and R-S-R spacer arrays displayed similar Nla Type I-C-mediated knockdown magnitude when targeting the *lacI* gene in an *Escherichia coli* platform (Fig. S1). This result indicates that the minimal spacer array is sufficient for knockdown with this system.

### Development of a way to efficiently reprogram the spacer(s)

To allow the efficient reprogramming of the gene-specific spacer/guide sequence, we designed a new vector called the Spacer Switch Plasmid containing ~950 bp of the *lctP* gene and ~100 bp of the *cas7* gene to allow replacing of the existing CRISPR array with a new one (Fig. 1B). Flanked by these homology arm regions are a chloramphenicol resistance cassette and the CRISPR array with new spacer(s). Selection for transformants on erythromycin and chloramphenicol replaces the resident spacer with the new spacer(s) (Fig. 1C). Transformation of this spacer switch plasmid occurs at an efficiency of 2 × 10^−3^ chloramphenicol-resistant transformants/CFU. We can also transfer the gDNA from a strain carrying the CRISPRi locus to any other Gc strain via DNA transformation, since using chromosomal DNA results in an efficiency of 2 × 10^−2^ erythromycin-resistant transformants/CFU, which allows for general use of the CRISPRi system in Gc.

### CRISPR interference enables targeted knockdown of Gc gene expression

To determine whether our CRISPRi platform can be used to knockdown a highly expressed gene, we programmed NlaCascade to target the *opaD* gene in an OpaD constitutively expressing, non-variable strain (OpaD_nv_) (40). The *opaD* gene is 1 of 11 *opa* loci that, in total, encode 7–9 unique Opa proteins. The *opa* genes are non-essential and all independently phase variable due to pentameric 5′CTCTT′3 repeats within the signal sequence of the open reading frame (ORF), which promote slipped-strand mispairing during DNA replication (41). These frameshifts result in the independent “on” or “off” expression of each Opa variant. The OpaD_nv_ strain was generated in a genetic background with in-frame, markerless deletions for all 11 *opa* gene loci (40). In this background, a full-length *opaD* gene with point mutations in the pentameric repeat region that prevents phase variation was introduced, resulting in the constitutively “on” expression of OpaD. OpaD expression imparts an opaque, dark-hued colony morphology (39).

**Fig 2:**
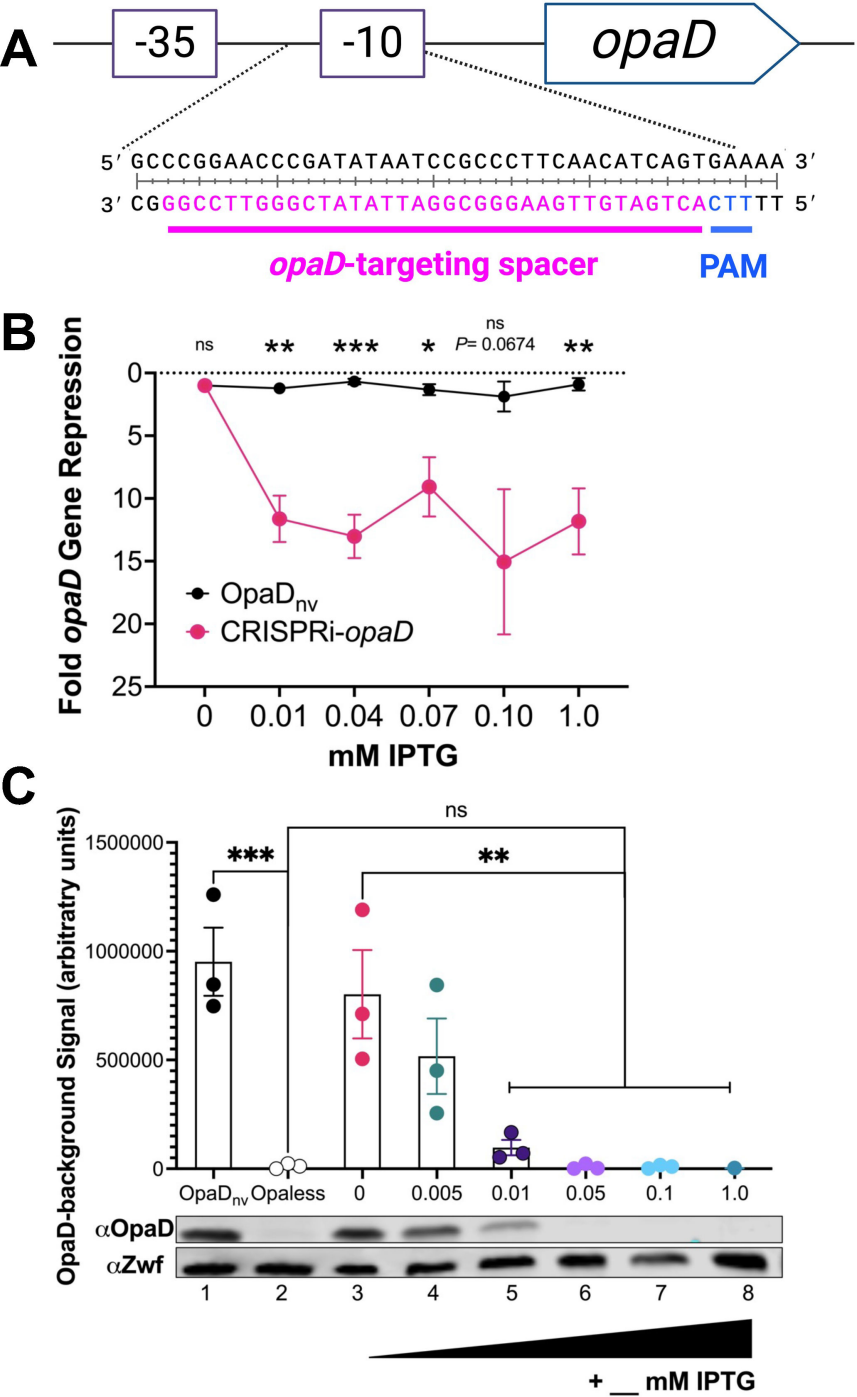
Analysis of CRISPRi-*opaD* regulation of OpaD expression. (**A**) Cartoon showing the *opaD*-targeting spacer sequence (pink) and PAM site (blue) relative to the −35 and −10 promoter regions (purple boxes) upstream of the *opaD* gene. (**B**) qRT-PCR analysis of *opaD* transcripts relative to the levels of *rmp* transcript. ΔΔ*C_T_* compared to each strain’s growth on 0.0 mM IPTG is displayed. Data are averages for four independent replicates, each with three technical replicates. Significance was determined using a Student’s *t* test to compare the two strains at each IPTG concentration. For *opaD* transcript levels, **P = 0.0014 (0.01 mM IPTG); ***P = 0.0004 (0.04 mM IPTG); *P = 0.0180 (0.07 mM IPTG); **P = 0.0065 (1.0 mM IPTG). (**D**) Western blot analysis of OpaD protein expression. A representative blot is shown, and intensity measurements are averages of three independent replicates. Significance was determined using a one-way ordinary analysis of variance (ANOVA) (*P* < 0.0001) followed by Tukey’s multiple-comparison test. For OpaD band intensity, ***P = 0.0004 (OpaD_nv_ vs Opaless); **P = 0.0065 (0 mM vs 0.01 mM); **P = 0.0022 (0 mM vs 0.05 and 0.1 mM); **P = 0.002 (0 mM vs 1.0 mM). For panels B and C, error bars indicate the standard errors of the mean.

We identified an *opaD*-specific protospacer sequence 3′ to a 5′TTC′3 PAM site and is 40 bp upstream of the *opaD* start codon (Fig. 2A). This spacer sequence overlaps the −10 element of the *opaD* promoter (42) and is designed to bind to the coding strand relative to the *opaD* mRNA transcript. To quantify *opaD* gene repression, we performed quantitative reverse transcription-PCR (qRT-PCR) and found a corresponding change in *opaD* transcripts with an IPTG dose response (Fig. 2B). Relative to the parental strain, the CRISPRi-*opaD* strain displayed a significant (~10-fold) decrease in *opaD* transcript with growth on 0.01 mM IPTG, with repression magnitude leveling out at higher IPTG concentrations. IPTG had no effect on *opaD* expression in the parental strain. Furthermore, OpaD_nv_ strains carrying the CRISPRi machinery expressing a spacer targeting the replication origin region (*ori*), a spacer that does not target the Gc chromosome (non-targeting), or no spacer (null) did not display any reductions in *opaD* gene expression (Fig. S2). These results show that the downregulation in *opaD* expression is specific for the targeting spacer sequence.

We used Western blot to analyze OpaD protein expression (Fig. 2C). As previously reported, the Opaless strain, deleted for all 11 *opa* loci, showed no OpaD expression. The CRISPRi-*opaD* strain displayed similar levels of OpaD expression as the parental strain following growth without IPTG. Addition of IPTG into the growth medium slightly decreased OpaD expression to intermediate levels at 0.005 mM IPTG but significantly decreased protein levels at 0.01 mM IPTG. No OpaD expression was detected at 0.05 mM, 0.1 mM, and 1.0 mM IPTG. These data show that this Type I-C CRISPRi system can be used to effectively knockdown expression of a highly expressed protein.

### Tunable modulation of Gc and polymorphonuclear leukocyte (PMN) interactions via OpaD CRISPRi

**Fig 3:**
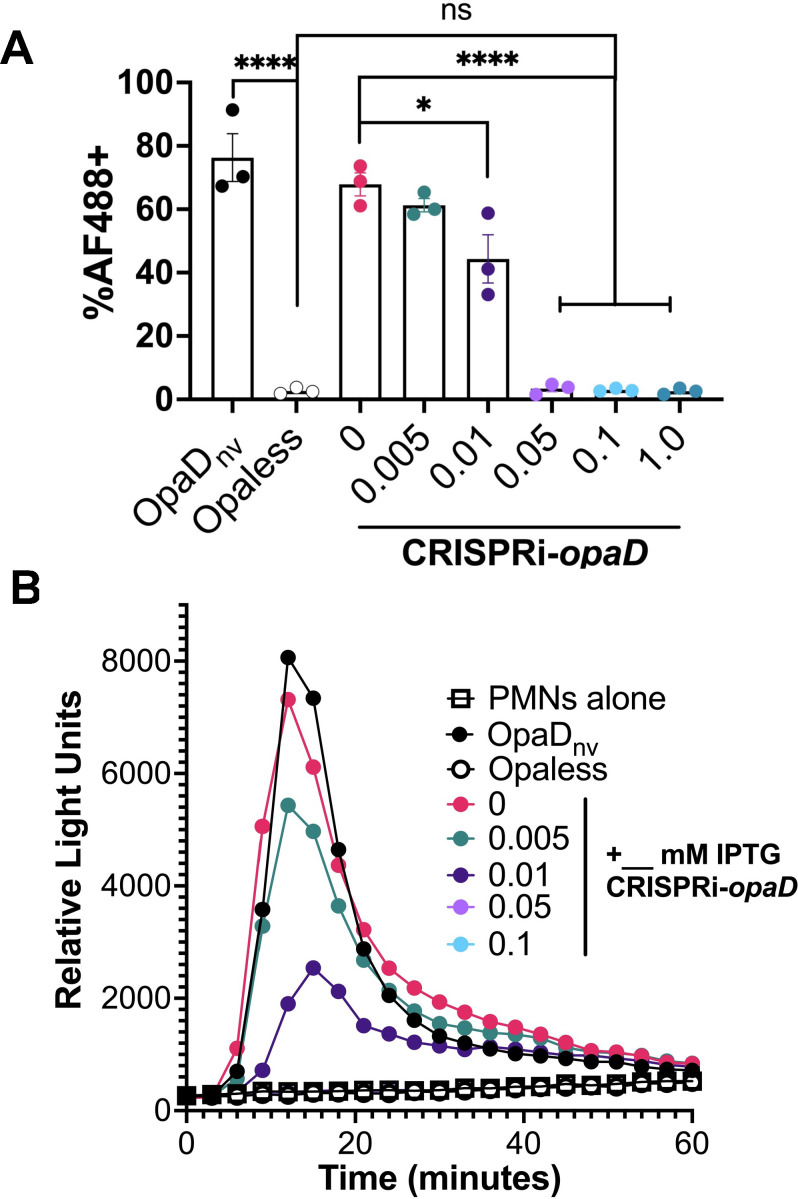
The Type I-C CRISPRi system modulates OpaD-dependent Gc-PMN interactions. (**A**) Flow cytometry-based N-CEACAM1 binding assay. CEACAM1 binding was measured as percentage of AF488-positive bacteria. Data are averages for three independent biological replicates. Statistical significance was determined using a one-way ANOVA (*P* < 0.0001) followed by Tukey’s multiple-comparison test. ****P < 0.0001; *P = 0.025. Error bars represent standard error of the means. (**B**) Luminol-dependent chemiluminescence assay using luminol chemiluminescence (relative light units) as a proxy for neutrophil ROS release. Data shown are one representative biological replicate.

Gc interactions with PMNs are central to disease pathogenesis, with patient-derived purulent exudate samples commonly containing mixtures of bacteria associated with PMNs (43, 44). These interactions are mediated through CEACAM-Opa contact, where OpaD mediates Gc binding to the PMN surface via CEACAM1 or CEACAM3 interaction to elicit an oxidative burst (45, 46). We quantified binding of OpaD expressing Gc to CEACAM1 using imaging flow cytometry in which 4′,6-diamidino-2-phenylindole (DAPI)-labeled Gc was incubated with the GST-tagged recombinant N-terminus of CEACAM1 (47). As previously shown, the OpaD_nv_ strain displayed higher binding than the Opaless strain (76% vs 2.7%) (Fig. 3A). At 0 mM IPTG, 68% of the CRISPRi-*opaD* cells were AF488 positive. The percentage of AF488-positive cells dropped slightly to 61% following growth on 0.005 mM IPTG but significantly to 44% on 0.01 mM IPTG. Following growth on 0.05, 0.1, and 1.0 mM IPTG, the binding decreased to approximately 3%. These results show that CRISPRi-mediated repression of *opaD* results in a tunable regulation of Gc-CEACAM1 binding.

We tested whether reducing OpaD protein expression in the CRISPRi-*opaD* strain would affect PMN reactive oxygen species (ROS) generation in an IPTG-dependent manner using a luminol-dependent chemiluminescent assay (48). Consistent with previous reports, OpaD_nv_ incubation with PMNs resulted in a strong ROS response, and the Opaless strain elicited no detectable ROS (Fig. 3B). PMN ROS generation in response to the CRISPRi-*opaD* strain was slightly diminished compared to the OpaD_nv_ strain in the absence of IPTG, suggesting that there may be a small repression even without IPTG. This small repression effect in the absence of IPTG was also apparent in Fig. 3A. Increasing the IPTG concentration to 0.005 mM, 0.01 mM, and 0.05 mM IPTG corresponded to a further reduction in ROS release. No chemiluminescence was detected when Gc was grown with more than 0.05 mM IPTG. These experiments provide further evidence that the Type I-C CRISPRi system can repress gene expression in a concentration-dependent manner and regulate PMN responses.

### CRISPRi enables multiplexed gene expression knockdown of all *opa* genes using a multiple-spacer CRISPR array

**Fig 4:**
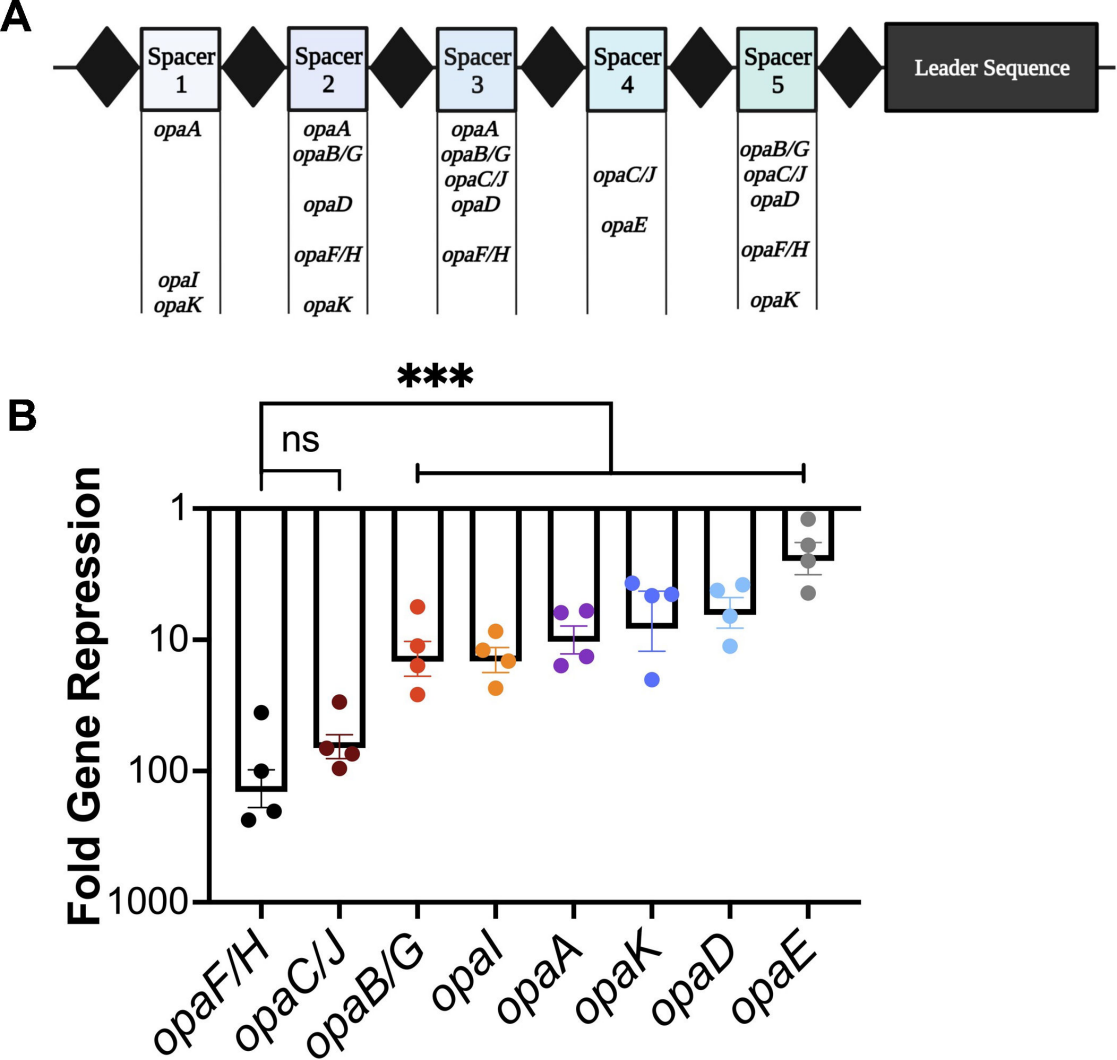
Gc-CRISPRi system can repress all 11 *opa* genes with a multi-spacer CRISPR array. (**A**) Schematic of the CRISPRi-*opa*MS array with the identity of each *opa* gene targeted. (**B**) qRT-PCR analysis of *opa* transcripts relative to the levels of *rmp* transcript. ΔΔ*C_T_* compared to growth on zero mM IPTG is displayed. Data are averages for four independent replicates, each with three technical replicates. Significance was determined using a one-way ANOVA (*P* < 0.0001) followed by Tukey’s multiple-comparison test. ***P = 0.0004 (*opaF/H* vs *opaB* and *opaI*), *P* = 0.0003 (*opaF/H* vs *opaA*), *P* = 0.0002 (*opaF/H* vs *opaK* and *opaD*), *P* = 0.0001 (*opaF/H* vs *opaE*). Error bars represent standard error of the means.

We next tested whether the Type I-C CRISPRi system can be used to simultaneously repress multiple genes from a single CRISPR array. We synthesized a spacer switch vector with five unique spacers predicted to together target all 11 *opa* genes (Fig. 4A), and each spacer targeted at least two *opa* genes. We introduced this array into strain N-1-60, as this strain encodes all 11 *opa* loci. This array was designated CRISPRi-*opa*MS (Opa multi-spacer).

Every *opa* gene was repressed in the CRISPRi-*opa*MS strain following growth on 1.0 mM IPTG (Fig. 4B). The range in gene repression varied from 140-fold for *opaF/H* to 2.5-fold for *opaE*, and the difference in repression was significantly greater in *opaF/H* compared to *opaB/G, opaI*, *opaA*, *opaK* and *opaD*, and *opaE*.

While all 11 *opa* genes are transcribed (41), there are differences in transcript abundance (49). We postulated that the range in CRISPRi-mediated *opa* gene repression could be explained by differences in basal *opa* gene expression. To calculate basal expression, we compared the cycle threshold (Ct) value of each *opa* gene to the housekeeping gene *rmp* at 0 mM IPTG. There was a partial correlation between the basal level of *opa* gene expression and knockdown magnitude (Fig. S3A). The *opaE* gene was both the lowest expressed and minimally repressed gene. The *opaF/H* genes showed the second highest level of expression and the highest level of repression. Similarly, the *opaA*, *opaB/*H, and *opaD* genes all showed intermediate levels of repression and intermediate levels of basal expression relative to the other *opa* genes. However, these correlations between basal gene expression and CRISPRi repression did not hold true for all the *opa* genes. The *opaI* gene showed the highest level of transcripts but was only the fourth most repressed gene, and the *opaK* gene had the third highest gene expression but showed the sixth lowest level of repression. Thus, basal gene expression is one of the parameters explaining the differences in gene repression by the CRISPRi-*opa*MS strain, but there are likely other factors as well.

We also hypothesized that the number of spacers targeting each *opa* gene in the CRISPRi-*opa*MS array could explain the range in *opa* gene repression magnitudes. We generated strains encoding the single targeting spacers derived from the CRISPRi-*opa*MS array (Fig. S3B) and compared the *opa* gene repression in these single-spacer constructs to the CRISPRi-*opa*MS five-spacer array (Fig. 4A). We first tested the three *opaF/H* spacers (Spacer 2, Spacer 3, and Spacer 5) individually and found the CRISPRi-*opa*MS strain displayed significantly greater repression magnitude than the single-spacer targeting constructs (Fig. S3C). While the Spacer 2 and Spacer 5 single targeting constructs resulted in 25- and 85-fold gene repression, respectively, the Spacer 3 construct only repressed *opaF/H* twofold. In contrast, repression by the full CRISPRi-*opa*MS array resulted in a 181-fold *opaF/H* repression. The other *opa* genes displayed similar repression patterns by the single-spacer constructs compared to the full CRISPRi-*opa*MS array. These results indicate that genes are repressed at greater magnitudes by multiple spacers than by a single targeting spacer and that some predicted targeting spacers are ineffective at repression. Overall, the difference in *opa* gene repression by the CRISPRi-*opaMS* strain is likely due to differences in basal gene expression and the number of targeting spacers per gene.

### Determining the release from CRISPRi repression

**Fig 5:**
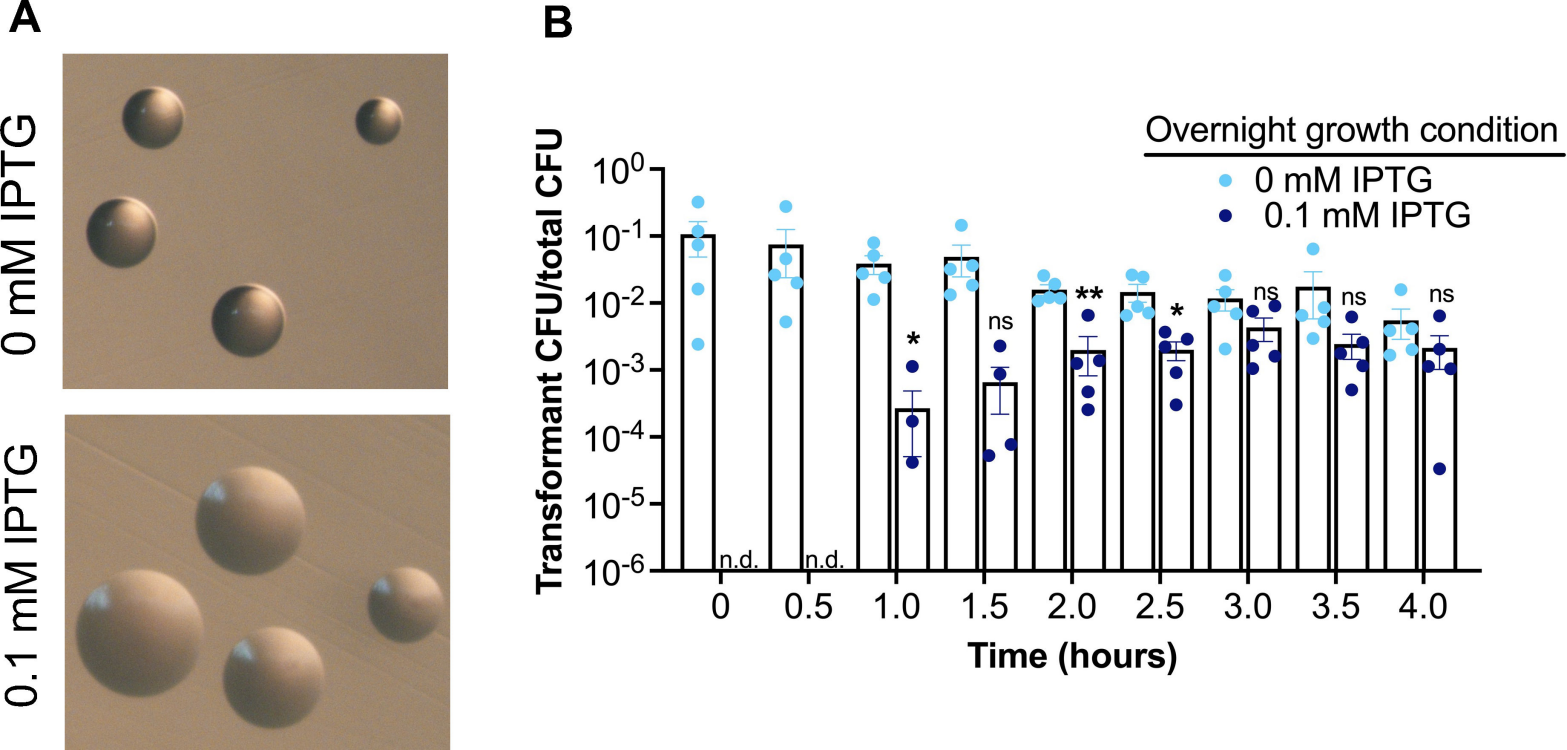
Timing of *pilE*-dependent competence restoration after IPTG depletion. (**A**) Colony morphology of CRISPRi-*pilE* strain plated in the absence or presence of 0.1 mM IPTG (bottom picture) and visualized with light microscopy. (**B**) DNA transformation efficiency assay with CRISPR-*pilE* plated overnight in GCB supplemented with 0.0 or 0.1 mM IPTG. Data are averages of five independent biological replicates, each with three technical replicates. Statistical significance was determined using the Student’s *t* test comparing the mean transformation efficiency at each timepoint. *P = 0.0127 (60 minutes); **P = 0.002 (120 minutes); *P = 0.0215 (150 minutes). Error bars represent standard errors of the means. N.d. indicates undetectable colonies.

We next quantified the duration of CRISPRi repression following IPTG removal. We targeted the *pilE* gene, which is not essential *in vitro* and encodes the major subunit of the type four pilus (Tfp). Expression of the Tfp is necessary for transformation competence, so we expected CRISPRi-mediated *pilE* repression to diminish DNA transformation (50–53). To control for Tfp antigenic variation, we performed these experiments in N-1-60, an FA1090 strain derivative defective for *pilE* antigenic variation and *pilC* phase variation (53). We used pilus-dependent colony morphology (54) to determine whether the CRISPRi-*pilE* strain yields a nonpiliated colony morphology when induced with IPTG. While uninduced CRISPRi-*pilE* colonies had a morphology consistent with piliated Gc (55), CRISPRi-*pilE* Gc plated on 0.1 mM IPTG showed colony morphology consistent with nonpiliated cells (Fig. 5A).

We used DNA transformation competence as a proxy for *pilE* expression status and measured the restoration of DNA competence over time following the removal of IPTG from the growth medium. To do this, we grew the CRISPRi-*pilE* strain overnight on gononoccal medium (GCB) plates containing 0 or 0.1 mM IPTG and transferred the bacteria from both conditions into IPTG-free liquid media. At 30-minute time points, we added pSY6, a plasmid containing a *gyrB* gene with a point mutation that confers nalidixic acid resistance once recombined with the chromosomal *gyrB* gene (56). Bacteria grown overnight without IPTG showed transformation of the mutant *gyrB* throughout the 4-hour time course (Fig. 5B). Gc grown overnight on 0.1 mM IPTG did not yield detectable numbers of Nal-resistant progeny at 0 and 30 minutes post IPTG removal. After 60 minutes of IPTG depletion, the Gc grown overnight on 0.1 mM IPTG began to display transformation competence, and the efficiency of transformation continued to increase at the 1.5-, 2.0-, and 2.5-hour time points. By 3 hours, the Gc achieved the same DNA transformation efficiency as the strain grown without IPTG. These results show that the Cascade stably bound to *pilE* promoter begins to turnover and lose repression ability of this target by 60 minutes.

### There are minimal growth defects associated with the ectopic expression of the Nla I-C CRISPRi machinery

**Fig 6:**
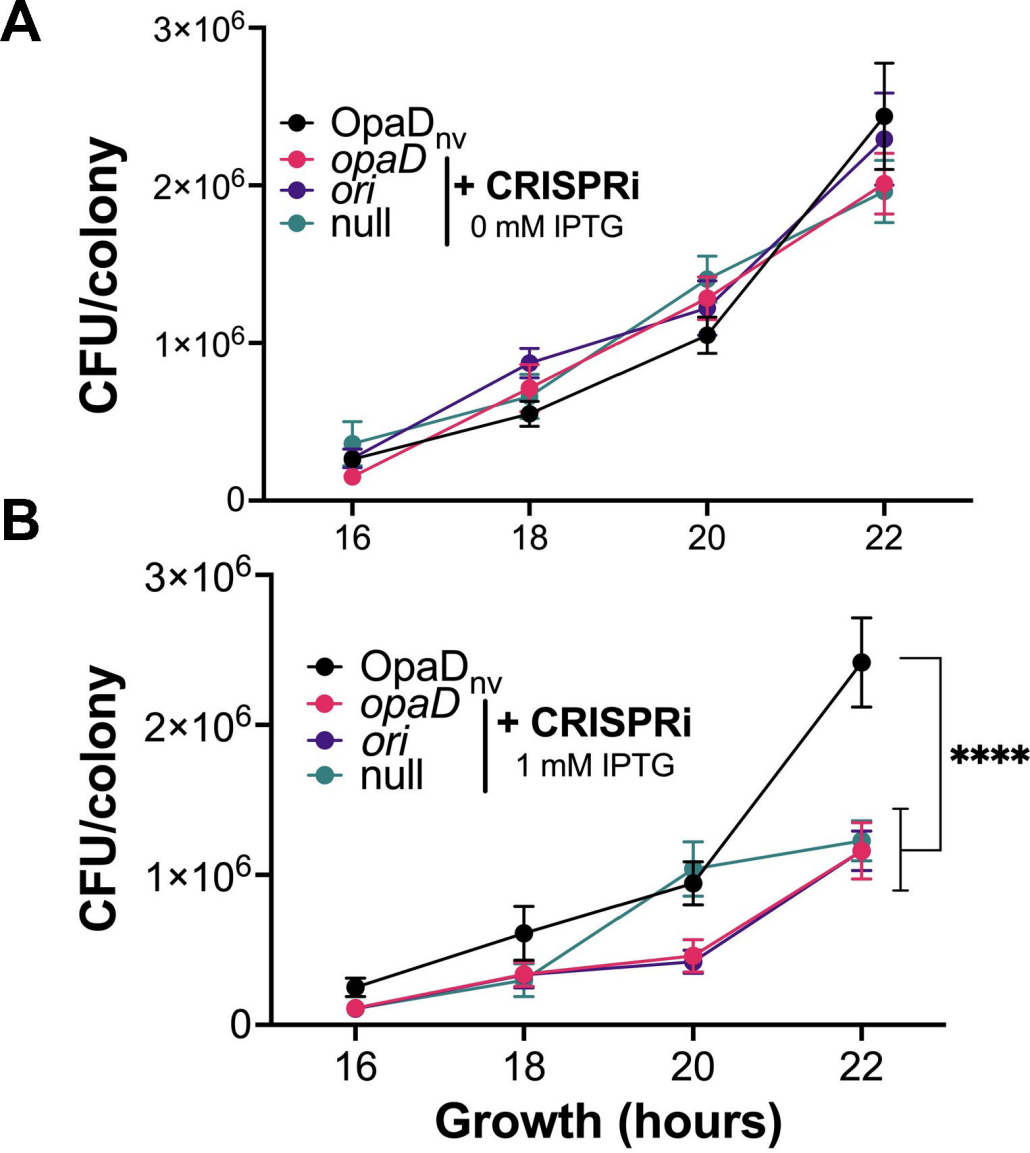
Analysis of the effect of CRISPRi expression on Gc fitness. Growth assay with CRISPRi-encoding Gc strains following overnight growth on solid GCB medium supplemented with (**A**) zero mM IPTG or (**B**) 1.0 mM IPTG. Data are averages of nine independent biological replicates. Significance for panel B was determined using two-way ordinary ANOVA *(P* < 0.0001) followed by Tukey’s multiple-comparison test. ***P < 0.0001. For both panels A and B, error bars represent standard errors of the mean. Abbreviation for origin-targeting spacer is *ori*; for no targeting spacer, null.

We determined the effect of CRISPRi expression on Gc fitness by measuring and comparing the CFU/colony for the OpaD_nv_ and the OpaD_nv_/CRISPRi-*opaD*, OpaD_nv_/CRISPRi-*ori*, and OpaD_nv_/CRISPRi-null strains on media with or without 1.0 mM IPTG. While there was no difference in growth among the strains grown with 0 mM IPTG (Fig. 6A), the addition of 1.0 mM IPTG to the growth medium slightly but significantly affected the growth of the CRISPRi-expressing strains (Fig. 6B). This effect occurred regardless of whether there was a spacer that directed the CRISPRi machinery to the genome. From these data, we conclude that the expression of the *cas* genes of Nla Cascade components causes a small fitness defect that should be accounted for when using Gc-CRISPRi with high IPTG concentrate.

### CRISPRi can be used to create conditionally lethal strains

**Fig 7:**
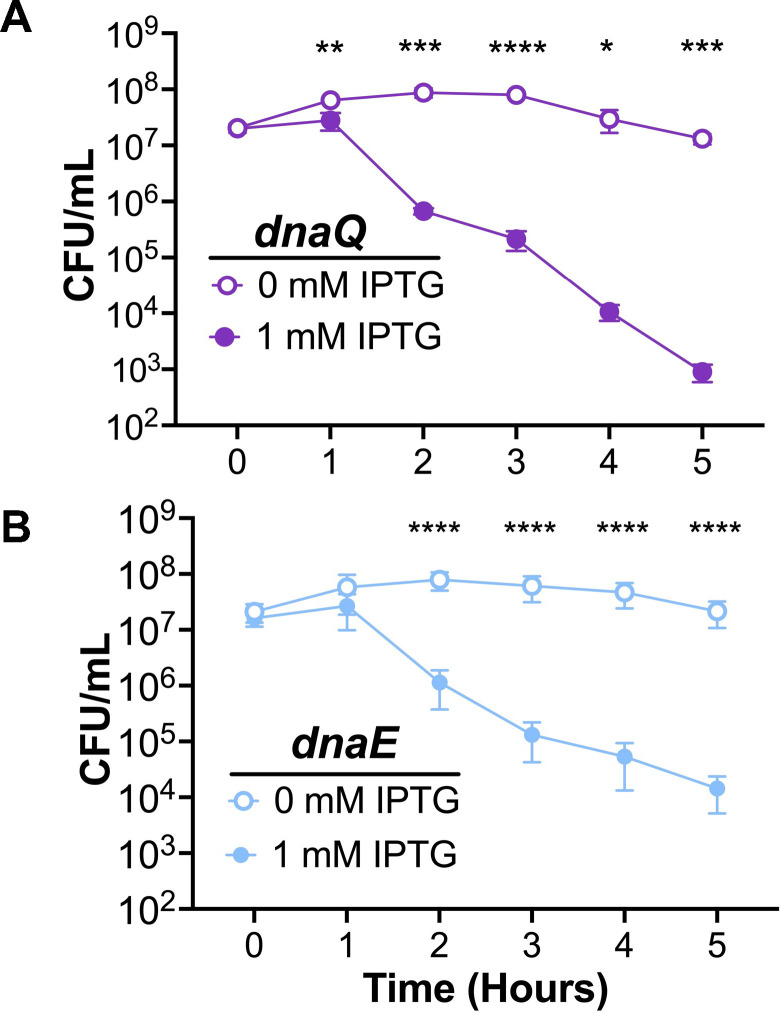
Gc-CRISPRi can create conditional lethality. Survival of Type I-C CRISPRi-encoding strains with (**A**) *dnaQ* and (**B**) *dnaE* targeting spacers. Data are averages of three independent replicates, each with three technical replicates. Significance was determined using a Student’s *t* test comparing the mean CFU/mL at each time point. For CRISPRi-*dnaQ*, **P = 0.0088 (1 hour); ***P = 0.0001 (2 hour); ****P < 0.0001 (3 hour); *P = 0.0357 (4 hour); ***P = 0.0002 (5 hour). For CRISPRi-*dnaE*, ****P < 0.0001. Error bars represent standard error of the means.

One important use for the Type I-C CRISPRi system would be to regulate the expression of essential genes. To test this possibility, we generated N-1-60 strains where the CRISPRi system targeted *dnaE* (CRISPRi-*dnaE*) and *dnaQ* (CRISPRi-*dnaQ*), essential components of the DNA polymerase III holoenzyme (57). When the CRISPRi-*dnaE* and CRISPRi-*dnaQ* strains were grown without IPTG, both strains displayed normal growth (Fig. 7). Growth with IPTG in the media resulted in small reduction in colony-forming units per milliliter (CFU/mL) for both strains within 1 hour of CRISPRi induction. The viability of both strains decreased significantly over a 5-hour time course, reflecting the turnover of the DNA polymerase III complex. These results indicate that the Type I-C CRISPRi system can be used to repress essential gene expression in Gc.

## DISCUSSION

The extant molecular toolbox for genetic manipulation in Gc is limited to generating individual knockouts and gene overexpression (58). We used the Cascade complex of the *N. lactamica*’s Type I-C CRISPR-Cas machinery to develop a minimal, IPTG-inducible CRISPRi system for Gc (38). We report that the Type I-C CRISPRi system efficiently knocks down gene expression in a gene-specific and tunable manner, and there are minimal fitness issues associated with the expression of this system in Gc. Importantly, we have developed an efficient way to change spacers, thus allowing one to reprogram the system to target any gene. Furthermore, the CRISPRi system can be used for PMN binding and response experiments. The repression effect is reversible, as gene expression begins to be restored at the 1-hour post-IPTG removal. This system can target multiple genes by simultaneously repressing the expression of all 11 *opa* genes within a single CRISPR array. Finally, by knocking down the expression of *dnaE* and *dnaQ*, we demonstrate that the Type I-C CRISPRi system can conditionally repress essential genes.

The Nla Type I-C CRISPRi system is capable of gene-specific knockdown with a minimal single-repeat, single-spacer CRISPR array. We originally designed this CRISPR array to facilitate site-directed mutagenesis to change the spacer sequence instead of using more cumbersome cloning methods due to the presence of multiple repeat sequences. To our knowledge, there has not been an engineered Type I CRISPR system using the R-S array. Most Type I-C systems encode spacers flanked by repeats, with each repeat forming stem-loop structures that are cleaved during crRNA maturation by Cas5 so that each spacer sequence is expressed into crRNA with a single stem-loop-bearing repeat (59–61). Cas5 will bind to both the 5′ and 3′ ends of the spacer-flanking repeat to cap the Cascade complex. The R-S array is still efficient for gene repression when programmed in *E. coli*, potentially suggesting that the Nla Type I-C system does not require Cas5 capping of the crRNA. Alternatively, Cas5-capped ends may be important for facilitating Cas3 nuclease activity but are unnecessary for stable DNA binding for gene repression. Whether this is unique to Nla Type I-C or more broadly conserved with other Type I-C CRISPR systems should be investigated.

We evaluated the stability of the Cascade complex by assaying for the return of transformation competence that is dependent on pilus expression following IPTG removal. We found that *pilE* gene expression is restored sufficiently to allow for competence within one doubling time (~60 minutes) following growth without IPTG. Conversely, we show by targeting *dnaQ* and *dnaE* that CRISPRi-mediated repression occurs within 1 hour of IPTG induction. We anticipate that the time for each target gene to be repressed and derepressed should be experimentally determined, for this timing may vary depending on gene product stability in each strain background.

The development of the Type I-C CRISPRi system serves as a useful system for assaying Gc physiology. Most infection experiments rely on strains carrying genetic mutations or knockouts, but these tools do not allow for the modulation in gene expression at a specific time of interest during *in vitro* or infection experiments. Importantly, we found that we can utilize the CRISPRi system to effectively knockdown colonization factors critical for interactions of Gc with PMNs, one of the cell types that Gc encounters in its human host (62). Fine-tuning gene expression at different stages of infection (i.e., host-cell attachment, internalization) provides a more nuanced understanding of a gene’s role that is difficult to determine via permanent genetic modifications alone. Furthermore, IPTG is highly diffusible, and the concentrations necessary for toxicity in eukaryotic cells (>50 mM) are far higher than the minimum concentrations required for effective CRISPRi gene repression (63–65). As such, the CRISPRi system can be used at specific points during PMN infection. The temporal knockdown of specific Gc genes may also be performed in humanized mice models of Gc infection, as IPTG is demonstrated to effectively modulate bacterial gene expression *in vivo* using IPTG-infused drinking water (66, 67).

The Type I-C CRISPRi system offers additional advantages for genetic manipulation in Gc that can be applicable to other bacterial model organisms. Gc are polyploid, containing approximately three copies of its genome in each coccal unit (68). Additional chromosomal copies may increase the possibility of generating merodiploids when engineering genetic knockouts, hindering the ability to delete certain genes (69, 70). CRISPRi can be used in place of genetic knockouts to ensure the complete repression of all copies of a gene. This may also be applied to the Gc-encoded cryptic and conjugative plasmid, which have varying copy numbers (71, 72). Similarly, Gc encodes several sets of paralogues (in addition to the 11 *opa* loci) that share significant stretches of sequence similarity, making it difficult to generate selectable mutations with unique antibiotic resistance cassettes in all these genes (40, 73). With CRISPRi, a single targeting spacer can efficiently knockdown expression in every genetic locus.

In addition to its use in Gc, the *N. lactamica* Type I-C CRISPRi could be a useful tool for gene repression in other bacteria. We have preliminary data suggesting that *Escherichia coli* carrying the Nla Type I-C CRISPRi delivery vector display repression capabilities (74). Furthermore, a previous study transferred a fully functional Type I-C CRISPR-Cas3 system from *Pseudomonas aeruginosa* into *E. coli*, *Pseudomonas syringae*, and the more genetically distinct *Klebsiella pneumoniae* to elicit efficient gene editing (75). Thus, it is likely that this system can be adapted to be functional in other bacteria besides Gc. This system has other properties that will make it useful in any bacterial system. The Nla Type I-C CRISPRi 5′TTT′3 and 5′TTC′3 PAM consensus sequences are short, allowing for the targeting of most—if not all—genes (24). Indeed, the N-1-60 Gc genome encodes about 12,200 PAM sites within 50 bp of each start codon, which is over six times the total number of genes (8,256 5′TTT′3 and 3,867 5′TTC′3 sites, 2,207 ORFs). This CRISPRi system may also help define spacers for bacteria with low GC genomic content, such as those from *Firmicutes*, *Gammaproteobacteria*, *Bacteroidetes*, and *Chlamydiae* phyla (76).

Negative effects to bacterial fitness could limit the versatility of the Nla CRISPRi system. However, we found that bacteria expressing the Type I-C CRISPRi Cas proteins displayed only minimal growth defects on solid medium, even at high IPTG concentrations. The small effect on Gc fitness was present regardless of whether there was an encoded spacer. While we measured growth on media supplemented with 1.0 mM IPTG, most experiments will not require such a high IPTG concentration, as we found that knockdown is effective at much lower IPTG levels. As such, the fitness defect will likely be inconsequential for most experimental setups. However, the degree of this defect can vary depending on the conditions and timing in which the bacteria are grown, so those wishing to use alternative media may need to experimentally determine this. This result contrasts with the expression of the Type II-nuclease dead Cas9 CRISPRi systems in different bacteria, where the expression must be fine tuned to prevent toxicity, even when using a non-targeting gRNA (77–79). While the expression of foreign proteins alone may be impacting bacterial fitness, this result does not exclude the possibility that the Cascade proteins are still non-specifically associated with the DNA during the search for a PAM and target sequence, as is seen by different Type I CRISPR systems in other bacteria (80, 81).

CRISPRi may aid in the discovery or evaluation of novel antimicrobials for increasingly resistant gonorrheal infections. To investigate potential gene targets, CRISPRi can be used in conjunction with or as an alternative to transposon-insertional sequencing (Tn-seq). Tn-seq involves generating random genome-wide transposon insertions where it is impossible to control exactly which loci are hit and how many hits a genome receives (82). Furthermore, shorter genes will be less likely to be hit. If an insufficiently saturating screen is performed, genes important for certain biological processes may be ignored. In contrast, CRISPRi allows for control of which genes or genetic elements are targeted (83). Unlike Tn-seq, CRISPRi repression magnitude is tunable, making it possible to study variation in gene essentiality under several experimental conditions (84–86). Tunable repression is especially vital in determining an antimicrobial’s mode of action if its target is essential. The repression of genes that encode the targets of small molecule antimicrobials will result in a more sensitive phenotype, allowing for the identification of antimicrobial targets (87, 88).

The reagents needed to use this system in Gc and other species are available by request to the corresponding author as are detailed protocols. We expect that this CRISPRi system will facilitate both basic and translational research into the pathogenesis and treatment of *N. gonorrhoeae*.

## MATERIALS AND METHODS

### Bacterial strains and growth conditions

Strains and their details are listed in Table S1. All strains are FA1090 derivatives, either in the OpaD_nv_ or N-1-60 backgrounds (40, 53). The OpaD_nv_ is a phase-locked OpaD constitutively expressing strain in an *opaA-K* deleted (Opaless) background (40). The N-1-60 strain contains multiple point mutations in the *pilE* G4 and is *pilC2* phase-locked “on” with the 1-81-S2 pilin variant (53). Strains were grown on Gonococcal (Gc) Medium Base (BD Difco) (36.25 g/L), agar (1.25 g/L), Kellogg Supplement I (22.2 mM glucose, 0.68 mM glutamine, 0.45 mM cocarboxylase), and Kellogg Supplement II [1.23 µM Fe(NO_3_)_3_] (54). Gc was grown at 37°C at 5% CO_2_. For liquid growth, Gc was grown in GCBL medium consisting of 15 g/L of Bacto protease peptone 3, 23 mM potassium phosphate dibasic, 7.35 mM potassium phosphate monobasic, and 17.11 mM sodium chloride, supplemented with Kellogg Supplement I and II and 5 mM sodium bicarbonate.

### Construction of Type I-C CRISPRi machinery delivery vector and CRISPR array

The *cas5*, *cas8c*, and *cas7* genes were amplified from *N. lactamica* strain ATCC23970 gDNA using primers containing PacI and PmeI restriction cut sites (Table S1) (38). The amplified fragment was inserted into the PacI/PmeI-digested pGCC4 plasmid (designated pYZ1342) to allow insertion of the CRISPRi locus into an intragenic site of the Gc chromosome (34). To generate the *opaD* CRISPR array, two oligo pairs oYZ2266 + oYZ2267 and oYZ2268 + oYZ2269 were annealed separately. These two annealed oligonucleotides were ligated to the BsaI-cut-CRISPRi delivery vector (pYZ1389) by T4 ligase (NEB). Transformants were screened by PCR and Sanger sequencing. Transformation efficiency of Gc was obtained by dividing the calculated CFU grown on selective media by the total number of bacteria.

#### Spacer sequence design

CRISPR spacers to guide the Cascade complex to the target site were designed as previously documented (38). A scan of the consensus Nla PAM sequence (5′TTC′3 and 5′TTT′3) for the gene of interest was performed within the 100 bp preceding and 100–200 base following the start codon (35, 89). The PAM is 5′ to the spacer sequence, and the spacer sequence consists of the 35 bp immediately downstream of the PAM. Previous reports suggest an ORF strand bias (template vs non-template strand) for the spacer-targeted strand in Type I CRISPR systems (35, 89, 90). Spacers designed to bind to the non-template strand were preferred when targeting the ORF, while no strand differentiation was used to design spacers upstream of the start codon.

#### Changing the targeting spacer

To change the targeting spacer, we designed a synthetic vector (Twist Biosciences) containing ~950 bp of homology to *lctP* and ~100 bp of homology to *cas7* in the Gc chromosome. The *lctP* and *cas7* homology regions flank the Cat 2–9 chloramphenicol resistance cassette (91), the new spacer, the Nla Type I-C CRISPR repeat sequence, and the leader sequence. The spacer switch plasmid was then used as a template for Q5 site-directed mutagenesis. Splicing by overhang extension was used to generate the new spacer and replace the existing spacer, with primers designed via NEBase Changer. We used the Q5 High-Fidelity polymerase (NEB) and subsequently treated this PCR product with T4 Kinase, Dpn1 (NEB), and T4 Ligase to ligate the PCR product and remove the PCR template. To create a CRISPR array with more than one targeting spacer and repeat sequence, spacer switch constructs were commercially synthesized by Genewiz. Transformants with the new spacer(s) were selected by plating on chloramphenicol (1.0 µg/mL) and erythromycin (1.0 µg/mL).

### CRISPR repression assay in *E. coli*

This assay was conducted as described earlier (92), except that the strains used do not contain the third pBAD-derived plasmid encoding anti-CRISPR genes. To construct the R-S-R SCANR spacer array, oligos oYZ3160 and oYZ3161 were ligated together in BsaI-cut pYZ1386, underwent PCR with primers oYZ22110 and oYZ2469, and then Gibson assembled into the Nla Cascade-containing pYZ1432 plasmid. The R-S SCANR array was constructed by Gibson assembling the oligo oYZ5355 into pYZ1432. To carry out the *E. coli* CRISPRi repression assay, an overnight culture of each final strain (3.0 mL Luria broth [LB] with 20 µg/mL chloramphenicol and 50 µg/mL kanamycin) was pelleted and resuspended in 1× PBS at OD_600_ of 1.0 and imaged using a ChemiDoc MP imager (Bio-Rad) on the Alexa488 channel for 0.036 s. These samples were then quantified for OD_600_-normalized GFP fluorescence using a microplate reader and plotted as described earlier (92).

### Analysis of Opa protein expression

SDS-PAGE and immunoblotting for Opa expression were conducted as described previously (93). Lysates were harvested from overnight lawns on GCB with various IPTG concentrations. Samples were normalized in PBS + 5 mM MgSO_4_ to equivalent optical densities and lysed in sample buffer containing 12 mM Tris pH 6.8, 5% glycerol, 0.4% SDS, 1% β-mercaptoethanol, and 0.02% bromophenol blue. Lysates were boiled for 5 minutes and sheared with a syringe and 25-gauge needle (BD 305122). Bands were resolved using pre-cast 4–20% Criterion TGX (Bio-Rad 5671094) protein gels and transferred in CAPS methanol buffer to polyvinylidene difluoride membranes. Blots were blocked in PBS + 0.5% Tween-20 then incubated with mouse 4B12 pan-Opa antibody (94) and detected using a secondary antibody, goat anti-mouse H + L conjugated to DyLight 800 (Thermo Fisher Scientific PISA510176) (94). The loading control was detection of the uniformly expressed cytoplasmic protein Zwf by rabbit antiserum (95) and secondary antibody goat anti-rabbit H + L conjugated to Alexa Fluor 680 (Invitrogen A21109). Fluorescent blots were imaged with a LI-COR Odyssey CL-X, and relative band intensities were quantified with Image Studio software.

### CEACAM1 binding of Opa expressing Gc

GST-tagged recombinant N-terminal domains of human CEACAM1 were purified and utilized as previously described (47). Gc was grown overnight with various IPTG concentrations, and 1 × 10^8^ bacteria were incubated with GST-tagged N-CEACAM1 for 30 minutes at 37°C with end-over-end rotation. Gc was washed and stained to detect the presence of CEACAM with a mouse anti-GST antibody p1A12 (BioLegend 640802) followed by an Alexa Fluor 488 conjugated goat anti-mouse antibody (Jackson ImmunoResearch 115-545-164). Gc was then resuspended in 2% paraformaldehyde containing DAPI DNA stain. To process and analyze N-CEACAM1 binding, ImageStream X Mk II imaging flow cytometer with INSPIRE and IDEAS v. 6.2 software was used. In each replicate, single-color Gc was used to create the respective data set’s compensation matrix. Bacteria were gated by DAPI positivity, singlet size, focused images, and Alexa Fluor 488 positivity.

### PMN isolation

All human subjects research was conducted in accordance with a protocol approved by the University of Virginia Institutional Review Board for Health Sciences Research (#13909). Neutrophils were isolated from venous blood collected from healthy human subjects via dextran sedimentation followed by a Ficoll gradient as described previously (96). PMNs were resuspended in Dulbecco’s phosphate-buffered saline (without calcium and magnesium; Thermo Fisher) with 0.1% dextrose and used within 2 hours after isolation.

### PMN oxidative burst

Neutrophils were resuspended in Morse’s defined medium containing 20 µM luminol (97). Gc was added at an MOI of 100, and the generation of reactive oxygen species was measured every 3 minutes for 1 hour via chemiluminescence using a Victor3 Wallac luminometer (98). For an additional negative control, uninfected neutrophils were used as before (40).

### RNA isolation and qRT-PCR analysis

Bacteria were grown overnight on GCB supplemented with or without IPTG (Sigma-Aldrich) for 18–20 hours and swabbed into Bacteria RNA Protect (Qiagen), pelleted, and frozen at −80°C. RNA was isolated via the RNeasy Mini Kit (Qiagen) and treated with DNase I (Promega) to remove contaminating gDNA. RNA was incubated with Random Hexamers (Promega) and subsequently reverse transcribed with the SuperScript III Reverse Transcriptase (Thermo Fisher). A no-RT control was made for each sample to ensure that there was minimal to no gDNA contamination. The qPCR primers used for this study were designed with the IDT Primer Design tool and are listed in Table S1. qPCR was performed using iTaq Universal SYBR Green Supermix (Bio-Rad) in a Bio-Rad iQ5 Real-Time Detection system. The Gc gene, *rmp*, was used as an internal control (99). Gene expression change was calculated using the 2^−ΔΔCT^ cycle threshold method (100). qRT-PCR experiments were conducted following the Minimal Information for Publications on Quantitative Real-Time PCR Experiments guidelines (101).

### Growth assays

For liquid medium growth, Gc was first grown overnight on solid media at 37°C at 5% CO_2_ for 19 hours, then swabbed, resuspended into 10-mL GCBL with Kellogg supplements and sodium bicarbonate, and grown for 2 hours. Cells were then diluted to an OD_550_ of 0.1 and grown for 2 hours to allow for consistent exponential growth. Following these two growth periods, the cells were diluted again to an OD_550_ of 0.1 and treated with or without IPTG. At 1-hour time points, samples were taken and diluted for CFU/mL calculation. For solid growth, bacteria were plated at equal colony densities on GCB supplemented with 0 or 1.0 mM IPTG for 19 hours. At 2-hour increments, three colonies per plate were separately isolated via Whatman disks (Sigma-Aldrich) and suspended into 0.5-mL GCBL. Suspensions were vortexed and then diluted for CFU/colony calculations.

### DNA transformation efficiency assay

The DNA transformation efficiency was measured as published previously (56). Bacteria were swabbed from overnight growth, resuspended into GCBL supplemented with 5 mM sodium bicarbonate, and diluted to an OD of 0.15. Each sample was supplemented with various IPTG concentrations and grown for 4 hours to allow for *pilE* repression. At 30-minute time intervals, 30 µL was sampled and incubated at 37°C for 20 minutes with 100 ng of the pSY6 plasmid, which is a DNA containing a *gyrB* mutation rendering nalidixic acid resistance upon transformation (56). After 20 minutes, the samples were treated with 1 U DNase I for 10 minutes to degrade non-transformed DNA. The bacteria were then grown in GCBL for 2 hours at 37°C before being serially diluted and plated for CFU/mL on GCB in the presence or absence of 2 µg/mL nalidixic acid (Dot Scientific).

### Statistics

In all experiments, excluding the oxidative burst assay, the reported results are depicted as the means ± standard errors for three independent experiments or more. Statistics were calculated using GraphPad Prism analysis software; a *P* value of >0.05 was considered significant. The Student’s *t* test and analysis of variance (one-way and two-way) were used as appropriate to determine significance, as specified in figure legends.

## Data Availability

Raw data for this study are deposited in the Northwestern Feinberg School of Medicine (FSM) data depository at https://doi.org/10.18131/47nrk-a3483. Spacer sequences are available in Table S1.

## SUPPLEMENTAL MATERIAL

Supplemental Materials mbio.03025-23-s0001.pdf Tables S1-S4; Figures S1-S3.

ASM does not own the copyrights to Supplemental Material that may be linked to, or accessed through, an article. The authors have granted ASM a non-exclusive, world-wide license to publish the Supplemental Material files. Please contact the corresponding author directly for reuse.
