# Supplementary material for: Development and implementation of a Type I-C CRISPR-based programmable repression system for Neisseria gonorrhoeae
Source: mBio. 2023 Dec 21;15(2):e03025-23. doi: 10.1128/mbio.03025-23 (PMC10865793; doi:10.1128/mbio.03025-23)
Supplement: Supplemental Materials — Tables S1-S4; Figures S1-S3. [file mbio.03025-23-s0001.pdf]

Supplemental Table 1. Strains, oligonucleotides, plasmids, and spacer sequences used in this study.

Strain Name	Species	Strain	Description	Reference/ Source
OpaD$_{nv}$	*N. gonorrhoeae*	FA1090	OpaD-consitutitively expressed in opaless, G4-locked strain	40
Opaless	*N. gonorrhoeae*	FA1090	Opa deficient, G4-locked strain	40
N-1-60	*N. gonorrhoeae*	FA1090	G4 multisite mutation, PilC2 phase-locked on	39
TTC_R-S-R	*E. coli*	BL21A1	Encodes pYZ1431 (pPAM-SCANR-TTC PAM) and pYZ2708 (pCBAD33-NlaCAscade+NlaCR-PAM SCANR R-S-R spacer array)	
NGG_R-S-R	*E. coli*	BL21A1	Encodes pYZ1417 (pPAM-SCANR-NGG PAM target) and pYZ2708 (pCBAD33-NlaCAscade+NlaCR-PAM SCANR R-S-R spacer array)	
TTC_R-S	E. *coli*	BL21A1	Encodes pYZ1431(pPAM-SCANR-TTC PAM target) and pYZ2973 (pCBad33-NlaCascade+NlaCR-PAM SCANR R-S spacer array)	
NGG_R-S	E. *coli*	BL21A1	Encodes pYZ1417 (pPAM-SCANR-NGG PAM target) and pYZ2973 (pCBad33-NlaCascade+NlaCR-PAM SCANR R-S spacer array)	
TTC_R-BsaI-R	E. *coli*	BL21A1	Encodes pYZ1431(pPAM-SCANR-TTC PAM target) and pY2707 (pCBad33-NlaCascade+NlaCR-R-BsaI-R)	
NGG_R-BsaI-R	E. *coli*	BL21A1	Encodes pYZ1417 (pPAM-SCANR-NGG PAM target) and pY2707 (pCBad33-NlaCascade+NlaCR-R-BsaI-R)	

CRISPR-*opaD*	*N. gonorrhoeae*	FA1090	OpaD$_{nv}$ background with NIa Type I-C CRISPRi genetic construct, *opaD* targeting spacer	
CRISPRi- non-targeting	*N. gonorrhoeae*	FA1090	OpaD+nv background with Type I-C CRISPRi gene loci, non-targeting spacer	This study
CRISPRi-*null*	*N. gonorrhoeae*	FA1090	OpaD+nv background with Type I-C CRISPRi gene loci, no spacer	
CRISPRi-*ori*	*N. gonorrhoeae*	FA1090	OpaD+nv background with Type I-C CRISPRi gene loct, replication origin targeting spacer	
CRISPRi-*pilE*	*N. gonorrhoeae*	FA1090	N-1-60 genetic background with Type I-C CRISPRi gene loci, *pilE* targeting spacer	
CRISPRi-*opa* MS	*N. gonorrhoeae*	FA1090	N-1-60 genetic background with Type I-C CRISPRi gene loci, *opa* multi-spacer array	
CRISPRi-Spacer2	*N. gonorrhoeae*	FA1090	N-1-60 genetic background with Type I-C CRISPRi gene loci, spacer 2 from *opa* multi-spacer array	
CRISPRi-Spacer3	*N. gonorrhoeae*	FA1090	N-1-60 genetic background with Type I-C CRISPRi gene loci, spacer 3 from *opa* multi-spacer array	
CRISPRi-Spacer5	*N. gonorrhoeae*	FA1090	N-1-60 genetic background with Type I-C CRISPRi gene loci, spacer 5 from *opa* multi-spacer array	
CRISPRi-*dnaQ*	*N. gonorrhoeae*	FA1090	N-1-60 background with Type I-C CRISPRi genetic construct, *dnaQ* targeting spacer	
CRISPRi-*dnaE*	*N. gonorrhoeae*	FA1090	N-1-60 background with Type I-C CRISPRi genetic construct, *dnaE* targeting spacer	

Oligo Name	Sequence (5' to 3')	Description	Source
oYZ2092	ttacgaattcccggattaattaactatggaa attattaaggaggc	Primer with PacI site for amplification of Type I-C CRISPR machinery from *N. lactamica*	This Study
oYZ2093	cactagggccggccgtttaaactcagcctaa tttacgcaacag	Primer with PmeI site for amplification of Type I-C CRISPR machinery from *N. lactamica*	This Study
oYZ2266	AAACACTGATGTTGAAGGGCGGA TTATATCGGGTTCCGG TCAGCCGCCTCTAGG	Oligo containing part of OpaD spacer and CRISPR repeat	This Study
oYZ2267	CGGCTGTGTGTTGAAAC ACTGATGTTGAAGGGCGGATTAT ATCGGGTTCCGG	Oligo containing part of OpaD spacer and CRISPR repeat	This Study
oYZ2268	GCCGCCTAGAGGCGGCTGA CCGGAACCCGATATAATCCGCCCTT CAACATCAGT	Oligo containing part of OpaD spacer and CRISPR repeat	This Study
oYZ2269	CTGA CCGGAACCCGATATAATCCGCCCTT CAACATCAGT GTTTCAACACACA	Oligo containing part of OpaD spacer and CRISPR repeat	This Study
oYZ2419	ATAGGTTAATGTCAATTCAATTTTT AATGAACGGACGTCGGAATTGCC AGCTGG	AatII+PAM insert AS	46
oYZ2420	AAAAATAGGCGTCGAGGCCCTTTC GTCTTCACCTGTCGAGTGCAAAAC CTTTCG	NGG PAM insert Forw	This Study
oYZ2469	CCTGGAGATCCTTACTCGAGGGTC AGGTATGATTTAAATGGTC	XhoI+SR2 AS	46
oYZ3160	AAACTGTCGAGTGCAAAACCTTTC GCGGTATGGCATGAT	Nla PAM SCANR spacer-1 Forw	This study
oYZ3161	CTGAATCATGCCATACCGCGAAAG GTTTTGCACTCGACA	Nla PAM SCANR spacer-1 AS	
oYZ5355	TTCGCGGTATGGCATGATTTTTTTT TCTAGATATCGCTCAATACTGAC	XbaI+PAM SCANR Forw	
Rmp Sense	TATTCGTTGCATTGCTCGCTTCCG	qPCR sense primer for *rmp* gene	Stohl, E.A., Criss, A.K. and Seifert, H.S. (2005) The
Rmp Antisense	TCTACGCGACCTTGGCTTGCTTTA	qPCR antisense primer for *rmp* gene	
OpaD Sense	TGTCCGCCGTTTACGATTT	qPCR sense primer for *opaD* gene	
OpaD Antisense	GGCGGTAAGAGTACCTGTTATTT	qPCR antisense primer for *opaD* gene	

OpaA Sense	TAAGCCAAGTCCTATCGTACGAG	qPCR sense primer for *opaA* gene	transcriptome response of Neisseria gonorrhoeae to hydrogen peroxide reveals genes with previously uncharacterized roles in oxidative damage protection. *Mol Microbiol*, **58**, 520-532.
OpaA Antisense	CTTCGTGGGTTTTGAAGCGGGTG	qPCR antisense primer for *opaA* gene	
OpaB Sense	CAAGAATCAGAATAAGAGAG	qPCR sense primer for *opaB* gene	
OpaB Antisense	GTGTCTGACGTGTCCGTAG	qPCR antisense primer for opaB gene	
OpaC/J Sense	TCCACGCCGTTTCTTCTCTCGGC	qPCR sense primer for *opaC/J* gene	
OpaC/J antisense	GGCGCTCCGCCATCCTGACCGGC	qPCR antisense primer for *opaC/J* gene	
OpaKEF antisense	GTGTCTGACGTGTCCGTAG	qPCR antisense primer for *opaKEF* gene	
OpaK Sense	GATGGTACCTCTTCTAGCGGCCG	qPCR sense primer for *opaK* gene	
OpaE Sense	GGGAAGAAACGATAATAGCG	qPCR sense primer for *opaE* gene	
OpaF Sense	AAAGAAAACAATGGCAGCGG	qPCR sense primer for *opaF* gene	
OpaI Sense	ggaatgcgtacggatgtttct	qPCR sense primer for *opaI* gene	This Study
OpaI Antisense	cttacgcctacgagcacattac	qPCR antisense primer for *opaI* gene	

Plasmid Name	Description	Source	Purpose
pYZ1342	backbone for pYZ1385 cloning	This study	NlaCascade insert amplified from *N. lactamica* ATCC23970 gDNA by oYZ2092+2093, and gibson assembled into pGCC4 cut by PacI&PmeI
pYZ1385	backbone for pYZ1386 cloning	This study	oYZ2232 was gibson assembled into BsaI- & rSAP-treated pYZ1342
pYZ1386	backbone for pYZ1427 cloning	This study	A repeat-BsaI-repeat fragment amplified from pYZ2010 using oYZ2112+2241, and a CR promoter fragment amplified from N. lactamica ATCC23970 gDNA using oYZ2111+2110 using were inserted into PmeI-cut pYZ1385

pYZ1389	expressing crRNA opa spacer 1 for CRISPRi	This study	two annealed oligos pairs oYZ2266+2267 +oYZ2268+2269 simultaneous ligated into BsaI-cut-pYZ1386
pYZ1431	pPAM-SCANR-TTC PAM target	46	E. coli CRISPRi assay
pYZ1432	pCBad33-NlaCascade +NlaCR-PAM SCANR		Backbone for cloning pYZ2707
pYZ1417	PAM-SCANR-NGG PAM target	This study	Q5 mutagenesis on pYZ1431 using oligo pair oYZ2419+2420
pYZ2707	pCBad33-NlaCascade +NlaCR-R-BsaI-R	This study	The insert NlaCR R-BsaI-R cassette was amplified from pYZ1386 using oligos oYZ2110+2469, and then Gibson assembled into PmeI- and XhoI-cut pYZ1432

pYZ2708	pCBad33-NlaCascade+NlaCR-PAM SCANR spacer (R-S-R)	This study	Oligo pair oYZ3160+3161 was ligated into pYZ1386 via BsaI site. The ligation reaction was then used directly for PCR templation using primers oYZ2110+2469. The resulting insert fragment was Gibson assembled into PmeI and EcoRV cut pYZ1432.
pYZ2973	pCBad33-NlaCascade+NlaCR-PAM SCANR spacer R-S	This study	oYZ5355 was Gibson assembled into XbaI-cut pYZ1432

Spacer Name	Sequence (5' to 3')	PAM sequence
opaD	ACTGATGTTGAAGGGCGGATTATATCGGGTTCCGG	TTC
Non-targeting	TTACCTTCCACACCGGCTGGCAACAACCATTATCA	--
ori	aaaactctcctgatgcggctcttccgaacaaaagg	TTT
pilE	gtgccgattttatgttttgaggtgaaattttatgc	TTC
opa MS-Spacer 1	ctgaaataatcgcttaccgtgcttattttgccttt	TTT
opa MS-Spacer 2	ggcggcgtaggctaaatccgcctgcacatacgggc	TTC
opa MS-Spacer 3	gcgggctggattcattttcggctccttattcggtt	TTT
opaMS-Spacer 4	gcaccggctgcatcgggataatcgcgggtgatgtg	TTT
opa MS-Spacer 5	actgatgttgaagggcggattatatcgggttccgg	TTC
dnaQ	gtaaattgattatttttaagcaatgtatttttct	TTC
dnaE	AGATGGCGGTGCAGGTAATGCCGTTTCGGATTCAG	TTT

1A

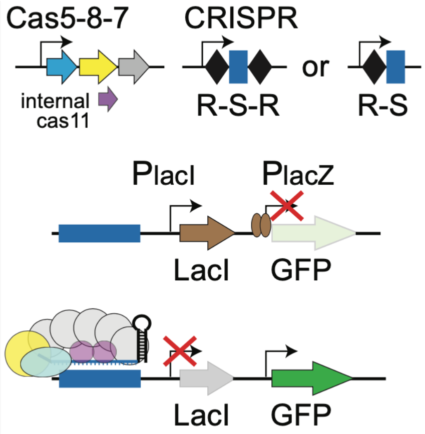
Cas5-8-7
CRISPR
or
internal
cas11
R-S-R
R-S
PlacI
PlacZ
LacI
GFP
LacI
GFP

1B

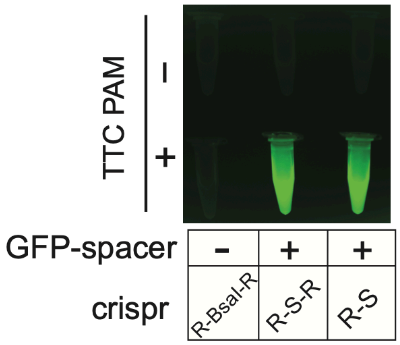
TTC PAM
–
+
GFP-spacer
–
+
+
crispr
R-BsaI-R
R-S-R
R-S

1C

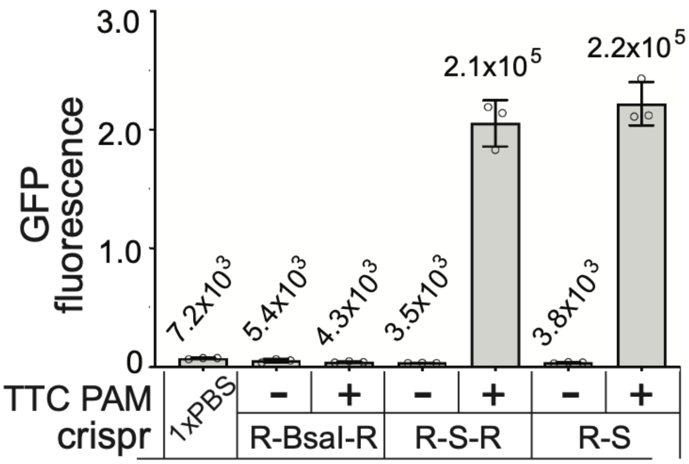
(10^5)
3.0
2.0
1.0
0
GFP fluorescence
7.2x10^3
5.4x10^3
4.3x10^3
3.5x10^3
2.1x10^5
3.8x10^3
2.2x10^5
TTC PAM
crispr
1xPBS
–
+
–
+
–
+
R-BsaI-R
R-S-R
R-S

Supplemental Fig 1. A repeat-spacer unit is sufficient for CRISPRi in *E. coli*.

A. Schematics of the CRISPRi based transcriptional assay in *E. coli*. Without a targeting Cascade, GFP reporter expression is blocked by LacI repressors (brown circles) produced from the upstream LacI cassette. NlaCascade binding to an engineered target site present in *lacI* promoter would lead to transcriptional blockage of LacI and in turn activates GFP expression. B. GFP image of OD_{600} normalized *E. coli* cultures from a representative experiment. Functionality in *E. coli* CRISPRi requires a GFP-targeting spacer, a TTC PAM next to the target site, but not the second repeat in CRISPR. For each strain, OD_{600}-normalized GFP fluorescence was quantified by microplate reader and plotted in C. Data shown are mean ± SD, n=3.

2

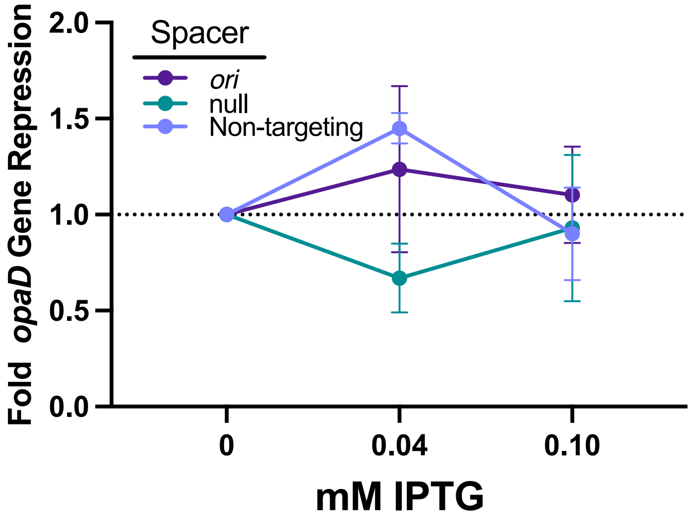

Supplemental Fig 2. The CRISPRi machinery targets *opaD* specifically.

qRT-PCR analysis of *opaD* transcripts relative to the levels of *rmp* transcript. $\Delta\Delta C_T$ compared to growth on 0 mM IPTG is displayed. Data are averages for three independent replicates, each with three technical replicates. Significance was determined using a two-way ANOVA (Effect of IPTG concentration: $F_{2,18}$=0.3169, *P*=0.7324; Effect of targeting-spacer: $F_{2, 18}$=1.132, *P*= 0.3443). Error bars represent standard error means. Abbreviations for origin-targeting spacer (*ori*), no targeting spacer (*null*), and non-genome targeting spacer (Non-targeting).

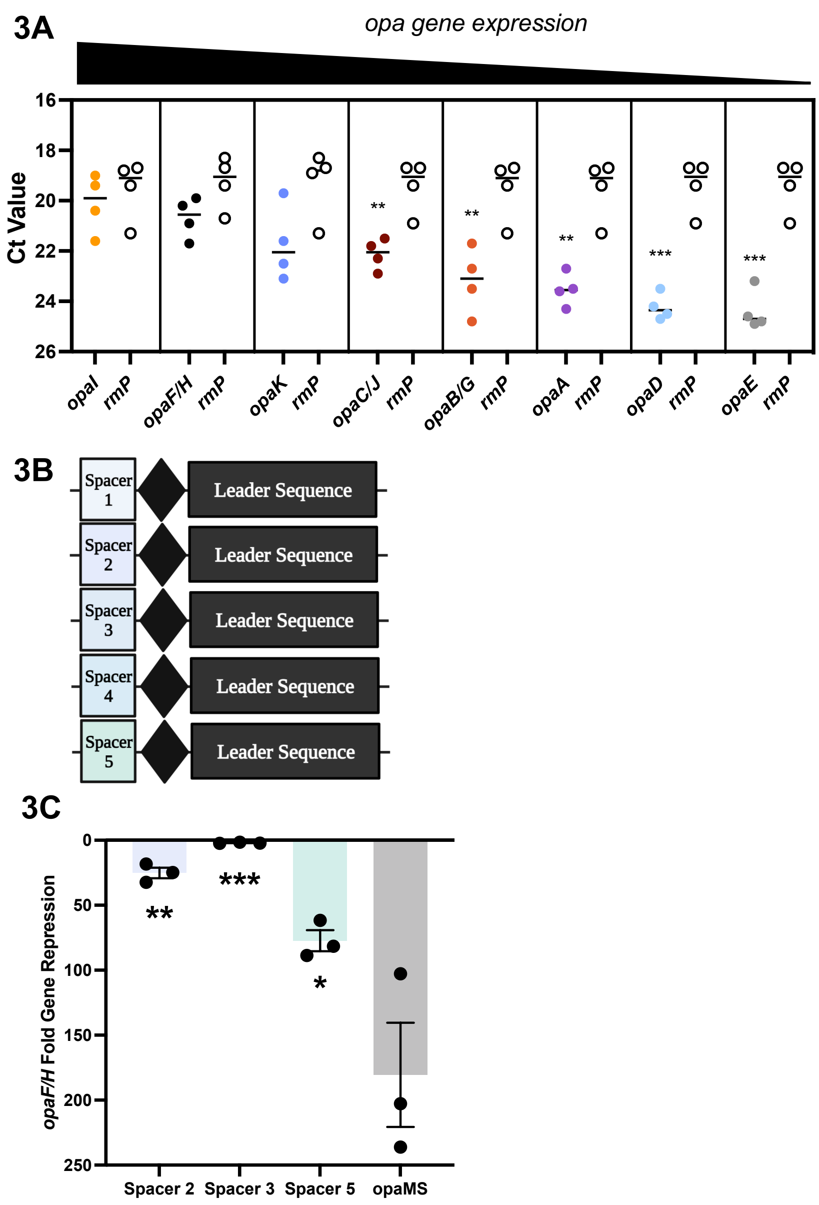

3A
opa gene expression
16
18
20
22
24
26
Ct Value
**
**
**
***
***
opaI
rmP
opaF/H
rmP
opaK
rmP
opaC/J
rmP
opaB/G
rmP
opaA
rmP
opaD
rmP
opaE
rmP
3B
Spacer 1
Leader Sequence
Spacer 2
Leader Sequence
Spacer 3
Leader Sequence
Spacer 4
Leader Sequence
Spacer 5
Leader Sequence
3C
0
50
100
150
200
250
opaF/H Fold Gene Repression
**
***
*
Spacer 2
Spacer 3
Spacer 5
opaMS

Supplemental Fig 3. Basal gene expression and quantity of gene-targeting spacers impact CRISPRi repression magnitude.

A. qRT-PCR analysis comparing the Ct value of each *opa* gene to its *rmp* value from RNA isolated from the CRISPRi-*opa*MS strain grown without IPTG. Data are averages of four independent biological replicates, each with three technical replicates. Significance was determined using Student's *t* test. Values shown are relative to each *opa*'s *rmp* Ct value. **, (*opaC/J*) *P*=0.0042; **, (*opaB/G*) *P*=0.0066; **, (*opaA*) *P*=0.0012; ***, (*opaD*) *P*=0.0002; ***, (*opaE*) *P*=0.0003. B. Schematic of the five single targeting spacers derived from the CRISPRi-*opa*MS array and cloned into individual strains. C. qRT-PCR analysis of *opaF/H* transcripts relative to the levels of *rmp* transcript. $\Delta\Delta C_T$ compared to each strain's growth without mM IPTG is displayed. Data are averages for three independent replicates, each with three technical replicates. Significance was determined using a one-way ANOVA (*P*=0.0026) followed by Dunnett's multiple comparisons test. Values shown are relative to the five spacer *opa*MS array. For *opaF/H* transcript, **, (Spacer 2) *P*=0.0018; *** (Spacer 3) *P*=0.0007; *, (Spacer 5) *P*=0.0187. Error bars represent standard error of the means.
